# Poor prognostic value of lymphovascular invasion for pT1 urothelial carcinoma with squamous differentiation in bladder cancer

**DOI:** 10.1038/srep27586

**Published:** 2016-06-09

**Authors:** Gang Li, Hualin Song, Jiaxin Wang, Yali Bao, Yuanjie Niu

**Affiliations:** 1 Department of Urology, The second hospital of Tianjin Medical University, Tianjin Institute of Urology, Tianjin, 300211, China; 2 Department of Maxillofacial and Otorhinolaryngology Oncology, Tianjin Medical University Cancer Institute and Hospital, National Clinical Research Center of Cancer, Key Laboratory of Cancer Prevention and Therapy, Tianjin, 300060, China; 3 Department of Pathology, The second Hospital of Tianjin Medical University, Tianjin, 300211, China

## Abstract

Lymphovascular invasion (LVI) is the primary and essential step in the systemic dissemination of cancer cells. The aim of our study was to assess the independent prognostic role of LVI for pT1 urothelial carcinoma with squamous differentiation in bladder cancer. We retrospectively analyzed the clinical and pathological information of 206 patients diagnosed pT1 urothelial carcinoma with squamous differentiation. Of the 206 patients, LVI was detected in 57 (27.6%) patients. The 5 year cancer specific survival (CSS) rates were 87.2% in LVI (−) and 52.4% in LVI (+) (p < 0.001). According to univariate analysis, tumor multiplicity, tumor size, recurrence and LVI were the prognostic factors associated with CSS. Additionally, tumor size and LVI significantly influenced the CSS in multivariate analysis. TURBT had shorter median CSS than RC in recurred patients with LVI (+). Our study suggested that LVI is an important predictor for survival of pT1 urothelial carcinoma with squamous differentiation. LVI positive status and tumor size ≥3 cm led to a higher risk of death. RC should be routinely performed in recurred LVI (+) bladder cancer patients of pT1 urothelial carcinoma with squamous differentiation.

Squamous differentiation is well known to occur in the bladder urothelial carcinoma and represents the most common form of mixed differentiation^1^,^2^,^3^,^4^. Lymphovascular invasion (LVI) is the primary and essential step in the systemic dissemination of cancer cells^5^. Numerous studies have been conducted to elucidate significant prognostic factors for LVI in bladder cancer^6^,^7^,^8^,^9^,^10^,^11^,^12^. Within the spectrum of urothelial malignancy, the significance of LVI has been well characterized for bladder cancer, but not for pT1 urothelial carcinoma with squamous differentiation in bladder cancer. The aim of our study was to assess the independent prognostic role of LVI for pT1 urothelial carcinoma with squamous differentiation in bladder cancer. To the best of our knowledge, this is the first study focusing on clinical significance of LVI for pT1 urothelial carcinoma with squamous differentiation.

## Patients and Methods

### Patients

We retrospectively analyzed the clinical and pathological information of 206 patients who were diagnosed as pT1 urothelial carcinoma with squamous differentiation from 2003 to 2014 in our institution. Clinical data was collected by a retrospective review of the medical records. We excluded patients with a history of previous urothelial carcinoma and concomitant upper tract urothelial carcinoma. All the patients underwent transurethral resection of bladder tumor (TURBT) and intravesical chemotherapy. All recurrent patients underwent re-TURBT or radical cystectomy (RC) according to individual histological grade. All the patients were divided into 2 groups by LVI or not, and the recurred patients was divided into 2 groups by TURBT and RC. Cystoscopy has been suggested to be given during the postoperative follow-up according to the European and US guidelines^13^. The prognostic factors were assessed including age, gender, tumor grade, tumor multiplicity, tumor size, recurrence, and LVI. In order to eliminate interference of influencing factor of bladder tumor grading we redo the analysis in subgroup of low-grade and high-grade tumor with or without LVI. The prognostic implications of these factors on cancer specific survival (CSS) rates were analyzed. All demographic and pathological variables were queried. Variables were evaluated for inconsistencies and data integrity. The pathologic stage was based on the 2009 Union for International Cancer Control (UICC) TNM staging system^4^. Grade was based on the 2004 World Health Organization (WHO) grading system for non-invasive urothelial neoplasia^2^.

### Pathology

All surgical specimens were submitted en bloc for pathological evaluation. Sectioning was performed on a case by case basis to provide adequate evaluation of grade and stage. Independent pathologic re-review of three representative slides from each patient was performed by two pathologists on all specimens to confirm reported pathologic findings and to confirm LVI status. The presence of intercellular bridges or keratinization was indicative of squamous differentiation. The presence of LVI in TURBT specimens was assessed using conventional hematoxylin and eosin (H&E) staining and immunohistochemical staining (IHC) markers against the lymphatic (D2-40) and vascular endothelium (CD 31)^14^,^15^. IHC assessment of LVI was performed on TURBT specimens of primary diagnosis. LVI was defined as the presence of the invasion of cancer cells into blood vessels or the lymphatic system or both and neoplastic cells within an endothelium-lined space.The criteria for diagnosing LVI did not change over the study period.

### Statistical methods

Cancer specific survival was considered from the day of surgery to the day of bladder cancer specific death. The chi-squared test and Student’s *t* –test were used to evaluate the association between categorical and continuous variables, respectively. The Kaplan-Meier method was used to calculate overall survival trends, and differences were assessed using the log-rank statistic. Univariate and multivariate Cox regression models were used to analyze overall survival after operation. All reported *P* values were two-sided, and a *P* value of ≤0.05 was considered to indicate statistical significance. Statistical analysis was performed with SPSS software (Version 22).

## Results

### Clinical characteristics

Clinical and pathological characteristics are listed in Table 1. Mean patient age was 67.2 years old. Of the 206 patients, 170 were males and 36 were females with a 4.7:1 male-to-female ratio. Of the study population of 206 patients, LVI was detected in 57 (27.6%) patients. LVI positivity was not significantly associated with gender (P = 0.693), age (P = 0.749), tumor multiplicity (P = 0.169) or tumor size (P = 0.967). However, high grade tumors were more common in LVI (+) than in LVI (−) (71.9% versus 28.1%, P < 0.001). Recurrence was appeared in 65 (31.6%) patients of which LVI (+) was 25 (43.9%) and LVI (−) was 40 (26.8%) (P = 0.019). The patients of recurrence underwent TURBT or RC that was carried out by the surgeons were listed in Table 2.

### Oncological outcome

**Figure 1:**
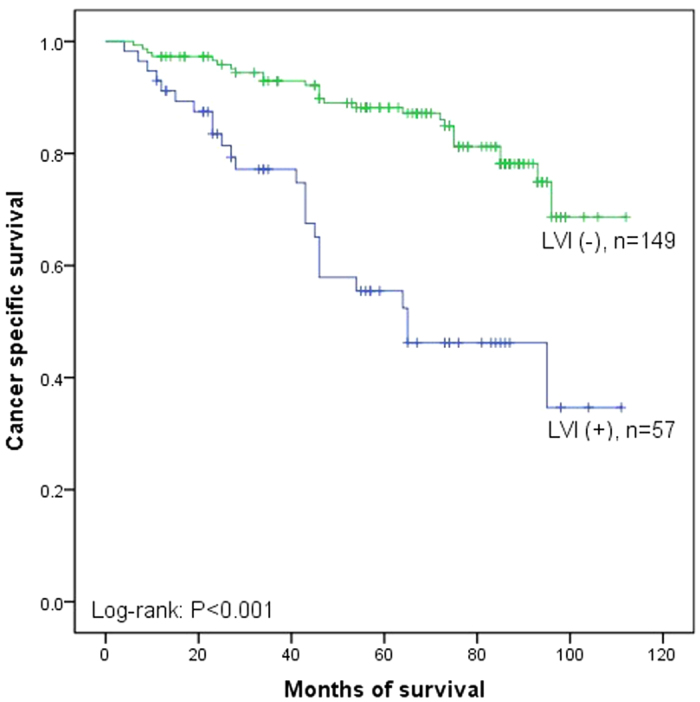
Kaplan–Meier curve of the cancer specific survival rates for LVI (−) and LVI (+) (P < 0.001).

**Figure 2:**
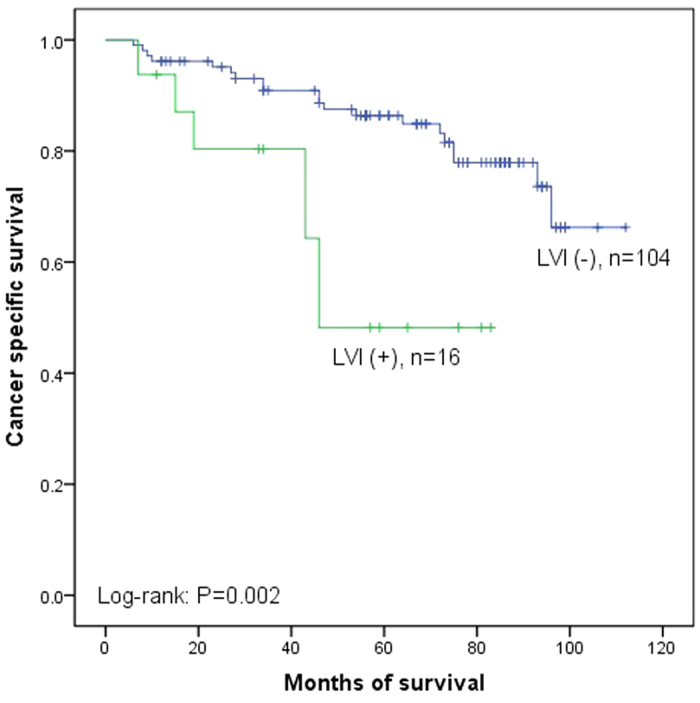
Kaplan–Meier curve of the cancer specific survival rates for LVI (−) and LVI (+) in subgroup of low-grade (P = 0.002).

**Figure 3:**
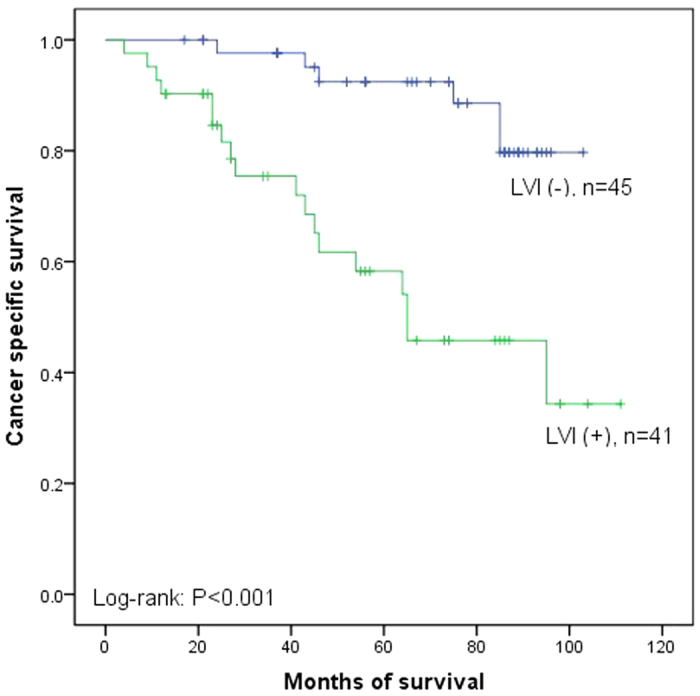
Kaplan–Meier curve of the cancer specific survival rates for LVI (−) and LVI (+) in subgroup of high-grade (P < 0.001).

Fifty-one patients out of 206 (24.3%) died of cancer metastasis during the median follow up of 62.2 months (range 4 to 112 months). The overall 5 year CSS rate was 78.3%. Moreover, the 5 year CSS rates were 87.2% in LVI (−) and 52.4% in LVI (+) (P < 0.001, Fig. 1). In subgroup of low-grade, the 5 year CSS rates were 86.4% in LVI (−) and 48.2% in LVI (+) (P = 0.002, Fig. 2) and in subgroup of high-grade, the 5 year CSS rates were 88.6% in LVI (−) and 54.1% in LVI (+) (P < 0.001, Fig. 3). Disease recurred in 65 (31.6%) patients. However, recurrence was more common in LVI (+) than in LVI (−) (43.9% versus 26.8%, P = 0.019). We used Cox proportional hazard analysis for further analysis (Table 3). According to the results of univariate analysis, we found that tumor multiplicity (hazard ratio (HR) 1.778, 95% confidence interval (CI) 1.020–3.099, P = 0.042), tumor size (HR 1.936, 95% CI 1.103–3.399, P = 0.021), recurrence (HR 1.988, 95% CI 1.130–3.496, P = 0.017), and LVI (HR 3.774, 95% CI 2.167–6.571, P < 0.001) were the prognostic factors associated with CSS. However, in multivariate Cox proportional hazard analysis, only tumor size (HR 2.942, 95% CI 1.557–5.562, P = 0.001) and LVI (HR 4.806, 95% CI 2.550–9.055, P < 0.001) significantly influenced the CSS.

**Figure 4:**
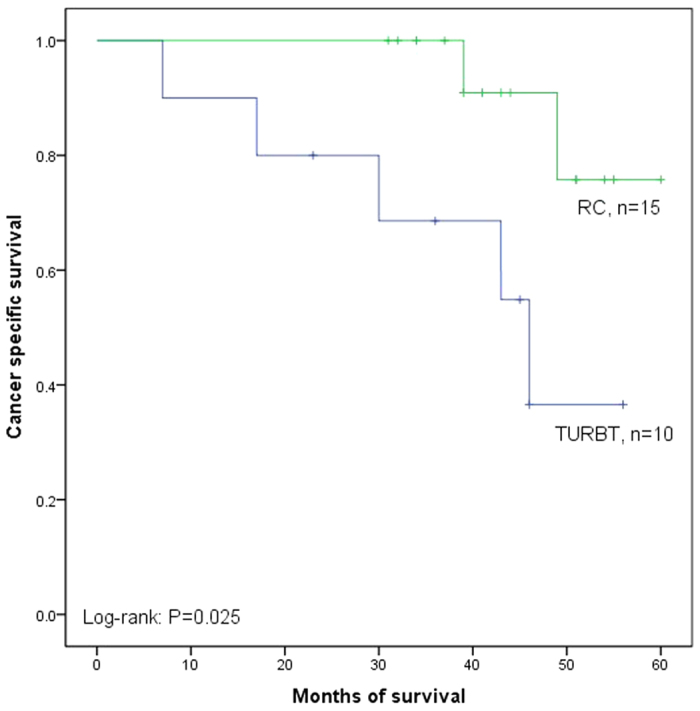
Kaplan–Meier curve of the cancer specific survival rates for TURBT and RC in LVI (+) (P = 0.025).

**Figure 5:**
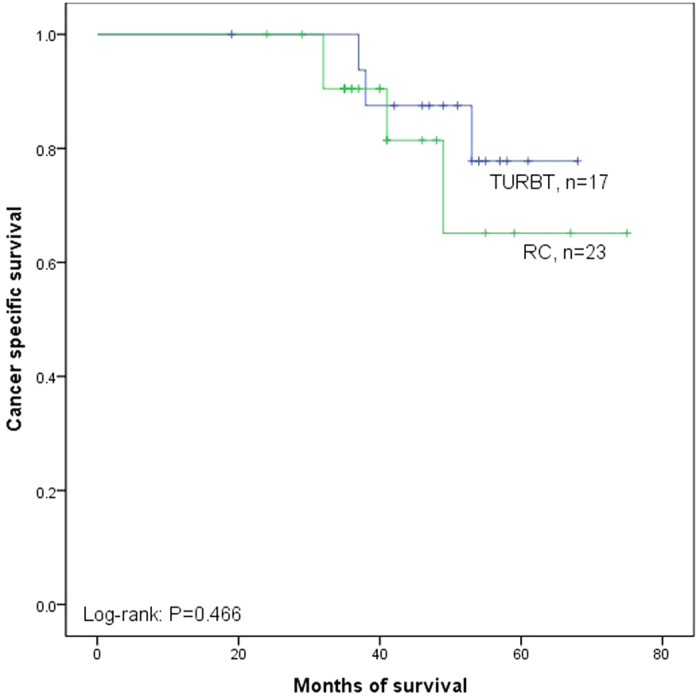
Kaplan–Meier curve of the cancer specific survival rates for TURBT and RC in LVI (−) (P = 0.466).

The Kaplan–Meier analysis was used to estimate CSS after recurrence stratified by TURBT versus RC in LVI (+) and LVI (−). Patients operated by TURBT had shorter median CSS duration than those operated by RC in LVI (+) (40.6 versus 56.4 months, P = 0.025, Fig. 4). However, no significant difference was observed between the two groups in LVI (−) (62.7 versus 63.6 months, P = 0.466, Fig. 5).

### Pathology and immunohistochemistry

**Figure 6:**
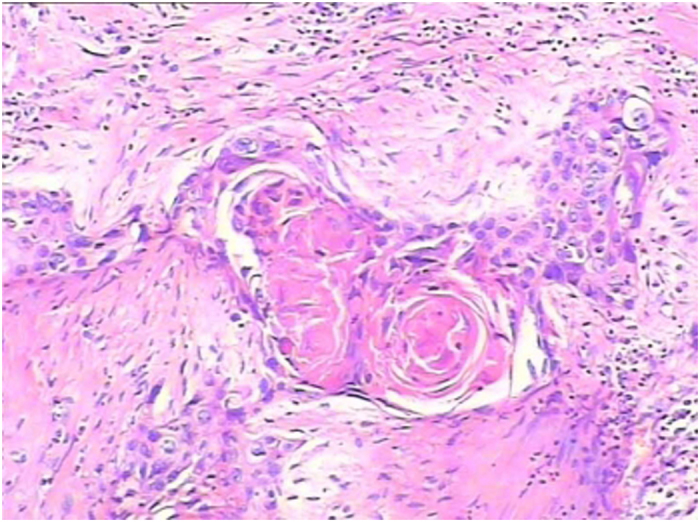
The component of tumor was considered to be squamous when intercellular bridges and/or keratinization were evident.

**Figure 7:**
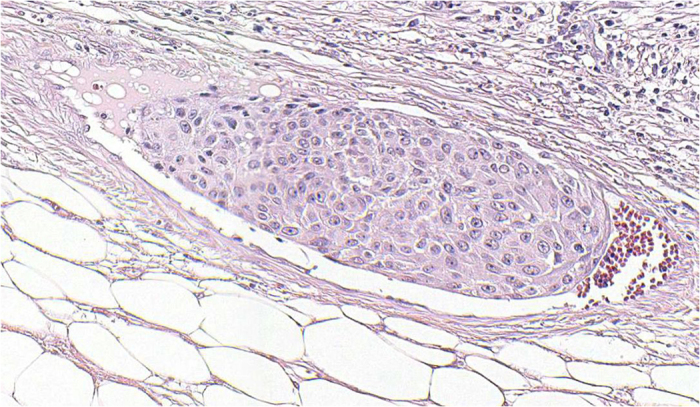
Stained sections in H&E were used to evaluate the presence of LVI.

**Figure 8:**
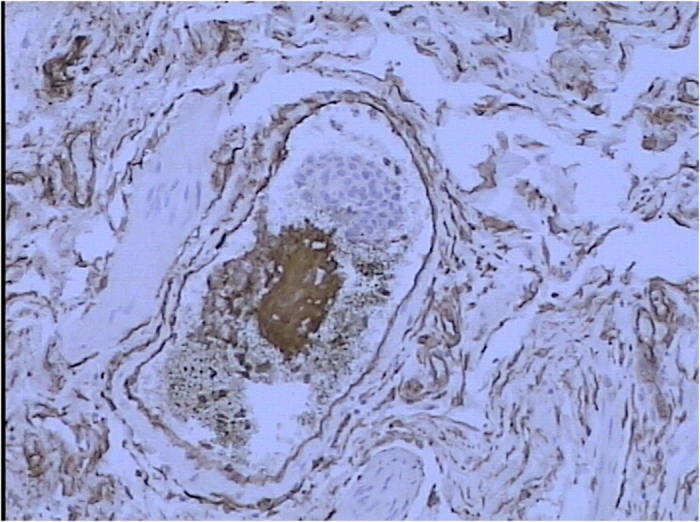
IHC stain in LVI (+) were positive for CD34.

Squamous differentiation was observed and confirmed by pathologists in all 206 cases. The component of tumor was considered to be squamous when intercellular bridges and/or keratinization were evident (Fig. 6). The tumors showed strands or nests of infiltrating tumor cells with large and medium sized nuclei, often with nucleolus, and a not clearly separated amphophilic or eosinophilic cytoplasmic background. Stained sections in H&E were used to evaluate the presence of LVI (Fig. 7), IHC staining of CD31, CD24, and D34 was then performed. IHC stain in these cases were positive for CD31, CD24 and CD34 (Fig. 8).

## Discussion

McDonald and Thompson reported the value of LVI as a criterion to assess the severity of urothelial bladder tumors for the first time^16^. Attention on the clinical significance of LVI in bladder cancer is growing, and a number of recent evidences have enhanced the significance of LVI for urothelial carcinoma of the bladder. Some papers indicated that LVI was an independent and significant prognostic factor for disease-specific survival^17^. Canter *et al*. analyzed the data from 356 patients treated with radical cystectomy by univariate analysis which found that the presence of LVI was a risk for overall, cancer-specific and recurrence-free survival (p < 0.0001)^7^. Cho *et al*., who conducted retrospective analyses of 118 patients reported that LVI, as an independent prognostic factor of progression and metastasis in pT1 bladder cancer, was significantly associated with disease recurrence^18^. The result was consistent with the study performed by Lopez and Angulo, in which multivariate analysis revealed that LVI was an independent prognostic factor in TURBT surgical specimens of T1 bladder cancer^19^.

However, there are short of data on the significance of LVI in patients of TURBT with urothelial carcinoma with squamous differentiation in bladder cancer, especially pT1 urothelial carcinoma of bladder. Our study showed further evidence suggesting that LVI was a pathological variable that might play an important role as a prognostic indicator in patients with pT1 urothelial carcinoma with squamous differentiation in bladder cancer. In our study, the presence of LVI was an independent prognostic factor related with disease survival (P < 0.001). In addition, there are hardly any data on the survival of the TURBT or RC in recurred patients in LVI for pT1 urothelial carcinoma with squamous differentiation in bladder cancer. Our finding indicated that recurred patients operated by TURBT had shorter median CSS duration than those operated by RC in LVI (+) (P = 0.025). However, no significant difference was observed between the two groups in LVI (−) (P = 0.466). According to the results, we suggest surgeons should operate RC routinely in recurred patients for pT1 urothelial carcinoma with squamous differentiation with LVI (+) in bladder cancer.

Shariat *et al*. conducted a retrospective review of 4257 radical cystectomy specimens and stratified the patients by LVI status and pathological stage^20^. Akdogan *et al*. demonstrated that ureteral UC had a higher recurrence rate and poorer survival rate than renal pelvic UC^21^. Our series found that LVI (HR 3.774, 95% CI 2.167–6.571, P < 0.001) was the independent prognostic factors associated with CSS in both univariate analysis and multivariate analyses. In addition, tumor multiplicity (HR 1.778, 95% CI 1.020–3.099, P = 0.042), tumor size (HR 1.936, 95% CI 1.103–3.399, P = 0.021), and recurrence (HR 1.988, 95% CI 1.130–3.496, P = 0.017) were the prognostic factors associated with CSS, too. However, in multivariate Cox proportional hazard analysis, only LVI (HR 4.806, 95% CI 2.550–9.055, P < 0.001) and tumor size (HR 2.942, 95% CI 1.557–5.562, P = 0.001) significantly influenced the CSS.

The presence of LVI in patients with newly diagnosed T1 urothelial carcinoma in bladder cancer is associated with decreased recurrence-free survival^18^. T1 urothelial carcinoma in bladder cancer accounts for about 30% of non-muscle-invasive bladder tumors, with varying degrees of aggressiveness and progression rates of up to 50%^22^,^23^. The lamina propria lying just beneath the epithelial lining is rich in lymphatic and blood vessels that allows for early lymphatic and hematogenous tumor spread^24^. In our study, the tumors showed strands or nests of infiltrating tumor cells with large and medium sized nuclei, often with nucleolus, and a not clearly separated amphophilic or eosinophilic cytoplasmic background. Stained sections in H&E were used to evaluate the presence of LVI. IHC stain in these cases were positive for CD31, CD24 and CD34.

## Conclusions

LVI is an important predictor for survival for pT1 urothelial carcinoma with squamous differentiation in bladder cancer. LVI positive status and tumor size ≥3 cm led to a higher risk of death which led to a higher mortality rate. Surgeons should operate RC routinely in recurred patients for pT1 urothelial carcinoma with squamous differentiation with LVI (+) in bladder cancer.

## Additional Information

**How to cite this article**: Li, G. *et al*. Poor prognostic value of lymphovascular invasion for pT1 urothelial carcinoma with squamous differentiation in bladder cancer. *Sci. Rep.* **6**, 27586; doi: 10.1038/srep27586 (2016).

## Figures and Tables

**Table 1:** Association of LVI with clinical and pathological characteristics. LVI, lymphovascular invasion, M, male, F, female.

**Characteristic**		**Total**	**LVI(−)**	**LVI(+)**	**P value**
Cases, n (%)		206 (100)	149 (72.3)	57 (27.6)	
Mean age (range), years		67.2 (29–92)	67.3 (29–92)	67.0 (33–85)	0.749
Gender, n (%)	M	170 (82.5)	122 (81.9)	48 (84.2)	0.693
	F	36 (17.5)	27 (18.1)	9 (15.8)	
	M:F	4.7:1	4.5:1	5.3:1	
Tumor grade, n (%)	Low	120 (58.3)	104 (69.8)	16 (28.1)	<0.001
	High	86 (41.7)	45 (30.2)	41 (71.9)	
Tumor multiplicity, n (%)	No	117 (56.8)	89 (59.7)	28 (49.1)	0.169
	Yes	89 (43.2)	60 (40.3)	29 (50.9)	
Tumor size (cm), n (%)	<3	145 (70.4)	105 (70.5)	40 (70.2)	0.967
	≥3	61 (29.6)	44 (29.5)	17 (29.8)	
Recurrence, n (%)	No	141 (68.4)	109 (73.2)	32 (56.1)	0.019
	Yes	65 (31.6)	40 (26.8)	25 (43.9)	

**Table 2:** The patients of recurrence underwent TURBT or RC.

	**LVI (−)**	**LVI (+)**	**Total**
TURBT	17	10	27
RC	23	15	38
Total	40	25	65

LVI, lymphovascular invasion, TURBT, transurethral resection of bladder tumor, RC, radical cystectomy.

**Table 3:** Univariate and multivariate Cox regression analyses predicting cancer specific survival.

**Variable**	**Univariate analysis**			**Multivariate analysis**		
	**HR**	**95% CI**	**P**	**HR**	**95% CI**	**P**
Age	1.007	0.983–1.032	0.555	—	—	—
Gender	0.442	0.175–1.112	0.083	—	—	—
Tumor grade	1.152	0.875–1.517	0.314	—	—	—
Tumor multiplicity	1.778	1.020–3.099	0.042	1.451	0.825–2.553	0.196
Tumor size	1.936	1.103–3.399	0.021	2.942	1.557–5.562	0.001
Recurrence	1.988	1.130–3.496	0.017	1.299	0.721–2.339	0.384
LVI	3.774	2.167–6.571	<0.001	4.806	2.550–9.055	<0.001

LVI, lymphovascular invasion, HR, hazard ratio, CI, confidence interval.
